# The efficacy of a lysine-based dendritic hydrogel does not differ from those of commercially available tissue sealants and adhesives: an ex vivo study

**DOI:** 10.1186/s12891-015-0573-7

**Published:** 2015-05-13

**Authors:** Juan C Villa-Camacho, Cynthia Ghobril, Lorenzo Anez-Bustillos, Mark W Grinstaff, Edward K Rodríguez, Ara Nazarian

**Affiliations:** Center for Advanced Orthopaedic Studies, Department of Orthopaedic Surgery, Beth Israel Deaconess Medical Center, Harvard Medical School, Boston, MA USA; Departments of Biomedical Engineering and Chemistry, Boston University, Boston, MA USA; Carl J Shapiro Department of Orthopaedic Surgery, Beth Israel Deaconess Medical Center, Harvard Medical School, Boston, MA USA

**Keywords:** Hemostasis, Trauma, Hydrogel, Sealant, Adhesive, Dendron

## Abstract

**Background:**

Hemostatic agents, tissue adhesives and sealants may contribute to a reduction in hemorrhage-associated morbidity and mortality. Towards this end, we have recently developed a lysine-based dendritic hydrogel (PEG-LysNH_2_) that can potentially be used in the management of severe trauma and/or intraoperative bleeding. As a first step in demonstrating the potential utility of this approach, our objective was to ascertain the ability of the PEG-LysNH_2_ to adhere to and seal injured tissues, as well as to maintain the seal under physiological conditions.

**Methods:**

The efficacy of the PEG-LysNH_2_ in sealing injured tissues was evaluated using an *ex-vivo* pressure testing system. A 2.5 mm incision was made on intact *ex-vivo* tissues and then sealed with the PEG-LysNH_2_. Application of the PEG-LysNH_2_ was followed by 1) step-wise pressure increase to a maximum of 250 mmHg and 2) fluctuating pressures, between 100–180 mmHg with a rate of 3 Hz, over a 24-hour period. The performance of the PEG-LysNH_2_ was compared to those of commercially available sealants and adhesives.

**Results:**

During gradual pressure increase, mean pressures at 30 seconds (P_30_) ranged between 206.36 - 220.17 mmHg for the sealants, and they were greater than control and suture groups (p < 0.01 and p = 0.013, respectively). Additionally, all products held under fluctuating pressures: mean pressures ranged between 135.20 - 160.09 mmHg, and there were no differences observed between groups (p = 0.96).

**Conclusions:**

The efficacy of the PEG-LysNH_2_ was significantly superior to conventional injury repair methods (sutures) and did not differ from those of commercially available products when sealing small incisions.

## Background

In the surgical setting, intraoperative bleeding that is unresponsive to conventional methods is associated with prolonged surgery times and significantly contributes to postoperative complications [1]. Topical hemostatic agents, sealants, and adhesives may be used during surgical procedures to achieve hemostasis when hemorrhage is not controlled by standard methods such as direct pressure, vessel ligation, and suturing [2]. These products physically adhere to damaged tissues and seal injured blood vessels to prevent further blood loss [3]. Additionally, specific agents may accelerate the production of a fibrin thrombus by activating the coagulation cascade [4].

We have recently developed a lysine-based dendritic hydrogel (PEG-LysNH_2_) that can potentially be used in the management of severe trauma and/or intraoperative bleeding [5]. As a first step in demonstrating the potential utility of the PEG-LysNH_2_, it was necessary to ascertain its ability to adhere to and seal injured tissues, as well as to maintain the seal under physiological conditions. We hypothesized that the PEG-LysNH_2_ would endure pressure testing in the absence of direct pressure by adhering to damaged tissue, and that its performance would be significantly better than that of conventional sutures and non-inferior to those of commercially available hemostatic agents, tissue adhesives and sealants.

## Methods

### Synthesis of the PEG-LysNH_2_

**Figure 1:**
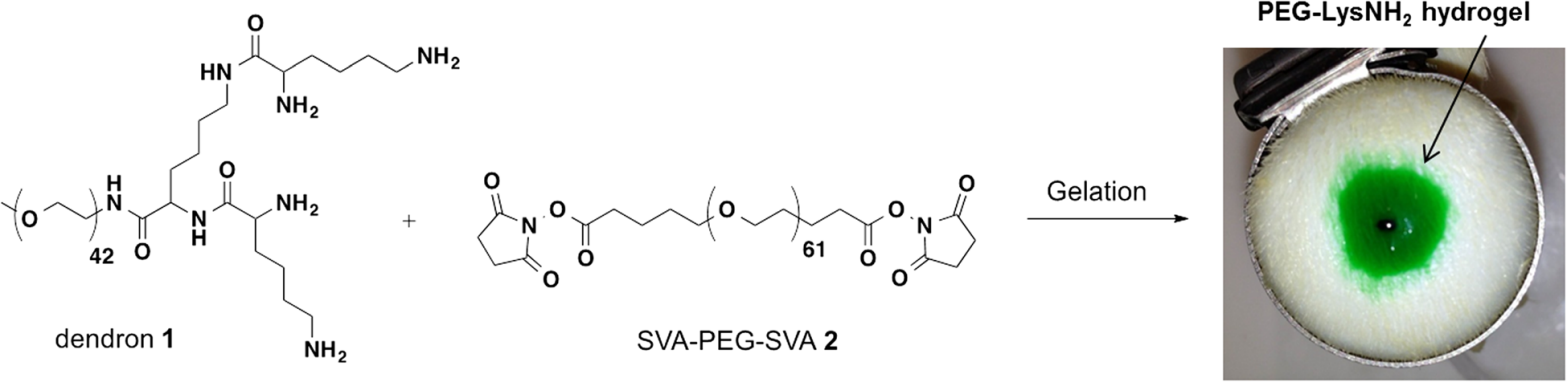
Formation of PEG-LysNH_2_ upon mixing of dendron **1** with SVA-PEG-SVA **2**. Hydrogel dyed with green food coloring.

The PEG-LysNH_2_ was synthesized using a previously reported procedure [5]. Briefly, a solution of pegylated lysine dendron **1** in borate buffer at pH 9 was reacted with a solution of poly(ethylene glycol disuccinimidyl valerate) of 3400 MW (**2**, SVA-PEG-SVA) in PBS buffer at pH 6.5 (Figure 1). The ratio of amine to SVA was 1:1, and the total concentration of polymer in solution was 30 wt%. A hydrophilic gel formed spontaneously within seconds upon mixing the two aqueous solutions.

### Rheological characterization

For each test material, cylindrical samples with a 9 mm diameter and 3 mm thickness were prepared in a precast Teflon mold and analyzed using 8 mm steel plate geometry. The mechanical strength and viscoelastic properties of the materials were investigated using dynamic rheological measurements and the frequency sweeps of all sealants and adhesives were measured at a frequency of 0.1 to 10 Hz with a controlled oscillatory stress of 50 Pa and at 20 °C (RA 1000, TA Instruments - New Castle, DE, USA) [5]. The shear storage (G’) and shear loss (G”) describe the elastic or solid-like and viscous or liquid-like characteristics, respectively, of a material. G’ values were reported as mean ± SEM (n = 3) for all sealants and adhesives, at 1 Hz of frequency and 50 Pa of oscillatory stress.

### Pressure testing

**Figure 2:**
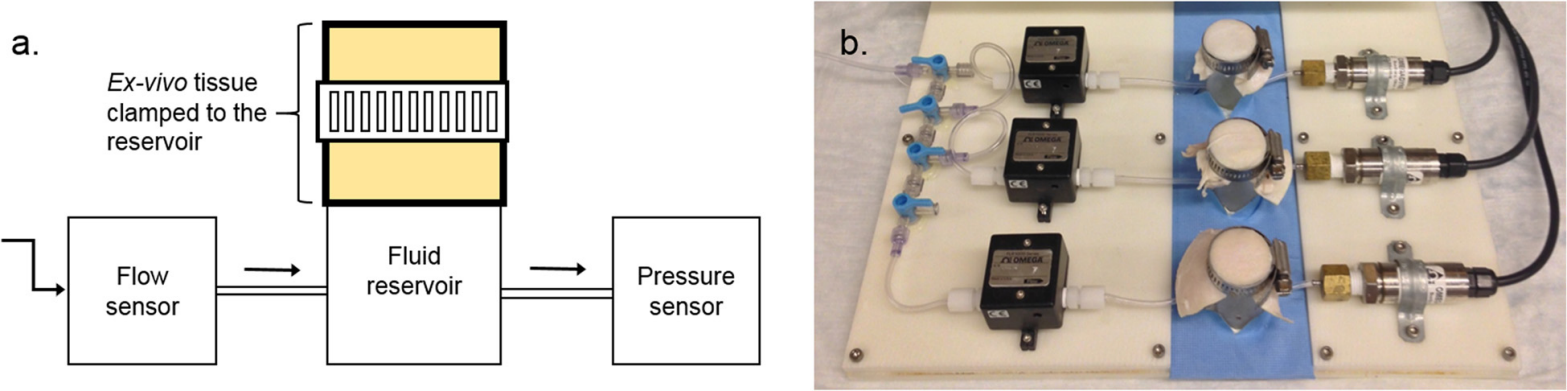
The failure testing device. **a.** Schematic representation of the testing device. Arrows indicate the direction of flow within the system; **b.** Photograph of the EVAH testing device. Saline solution was fed into the system with an infusor or a peristaltic pump (not shown).

The testing device consisted of a sensor assembly connected to a cylindrical reservoir (Figure 2). The sensor assembly contained a flow sensor (FLR-1007, Omega Engineering - Stamford, CT, USA) as well as a pressure sensor (PX-309, Omega Engineering - Stamford, CT, USA), which acquired data at a per-second rate and sent it to a data logger (DAQPRO-5300, Omega Engineering - Stamford, CT, USA). The sensor assembly was connected to the reservoir through polyvinyl chloride (PVC) pressure monitoring lines (MX561, Smiths Medical - Dublin, OH, USA) creating a closed system. The reservoir was lined with *ex-vivo* tissues (bovine aorta or murine skin) (Figure 2b), and 0.9% sodium chloride solution was fed into the system in either a continuous or pulsatile fashion - to simulate venous and arterial bleeding - using either a pressure infusor (Infusable, Vital Signs Inc. - Totowa, NJ, USA) or a peristaltic pump (SP04 L, Otto Huber GmbH - Böttingen, Germany).

We compared the efficacy of the PEG-LysNH_2_ to those of commercially available agents (Table 1). In order to comply with the original indications of the products, efficacy was tested on small wounds under dry conditions. Agents designed for vascular hemorrhage control were tested on bovine aortas, whereas topical agents were tested on murine skins. The PEG-LysNH_2_ was tested on both tissue types. All tissues used in the study were fresh frozen, preserved at −20°C and thawed for 30 minutes before testing. All products were applied directly on the wound in multiple thin layers with syringe or “gun” dispensers. Mixture of the products took place on the dispenser (Dermabond, Omnex, PEG-LysNH_2_), the dispenser tip (BioGlue) or directly on the wound surface (Evicel). Only one application was attempted in all trials.

**Table 1:** **Mechanism of action of the products used in the study**

**Agent**	**Brand Name**	**Category**	**Mechanism of action**
Glutaraldehyde cross-linked albumin	BioGlue	Sealant	Glutaraldehyde cross-links bovine albumin to cell proteins at wound site to form a tough scaffold [2].
Fibrin sealant (human)	Evicel	Sealant, hemostatic agent	Thrombin and fibrinogen mixed at site of application; thrombin cleaves fibrinogen to clot-forming fibrin [2].
Butyl lactoyl cyanoacrylate	Omnex	Adhesive	Liquid monomers that rapidly form polymers in the presence of water and thereby quickly glue surfaces together [2].
2-octyl cyanoacrylate	Dermabond	Adhesive	Liquid monomers that rapidly form polymers in the presence of water and thereby quickly glue surfaces together [2].
Lysine-based dendritic hydrogel (PEG-LysNH_2_)	Not available	Sealant	The cross-linked hydrogel forms an adhesive physical barrier on the wound’s surface [5] - hemostasis is not dependent on the clotting cascade.

### Resistance to failure

A 2.5 mm incision was made on the otherwise intact tissues and then sealed with the products (n = 3 per group). Three control groups were included in the tests: 1) intact tissue, 2) incised tissues without sealing and 3) incised tissues repaired with sutures (two vertical mattress suture stitches with an approximate bite size of 3 mm for the large bite and 1.5 mm for the small bite) using 4–0 polypropylene. After 10 minutes, pressure within the system was increased, in a stepwise fashion, by 40 mmHg every 5 seconds to a maximum of 250 mmHg, or until failure (noted by a sudden drop in pressure recordings, sudden rise in flow recordings, and visible leakage of saline through the wound). Mean pressure in the system at 30 seconds (P_30_) was calculated and compared between groups using one-way analysis of variance (ANOVA). Bonferroni corrected, two-tailed p values were used to establish statistically significant differences using α = 0.05 as the initial criterion. Relative standard error was calculated as an index of measurement reproducibility. Power calculations which indicated that 3 experiments would provide 88.1% power (β = 0.881) to detect a difference of 25 mmHg between treatment groups (δ = 25 mmHg) using the standard deviation of P_30_ intact tissue (σ = 7.31 mmHg).

### Endurance

We assessed the capacity of the products to hold and withstand fluctuating pressures analogous to human arterial pressures. A 2.5 mm incision was made on the otherwise intact tissue and then sealed with the products (n = 3 per group). A control group, of intact tissues only (without incisions) was included in this experimental phase. After 10 minutes, the system was subjected to fluctuating pressures (between 100 and 180 mmHg) with a rate of three cycles per second (Hz). Mean pressures were calculated for a 24-hour period and compared between groups using one-way ANOVA. Bonferroni corrected, two-tailed p values were used to establish statistically significant differences using α = 0.05 as the initial criterion.

## Results

### Use and rheological properties

**Figure 3:**
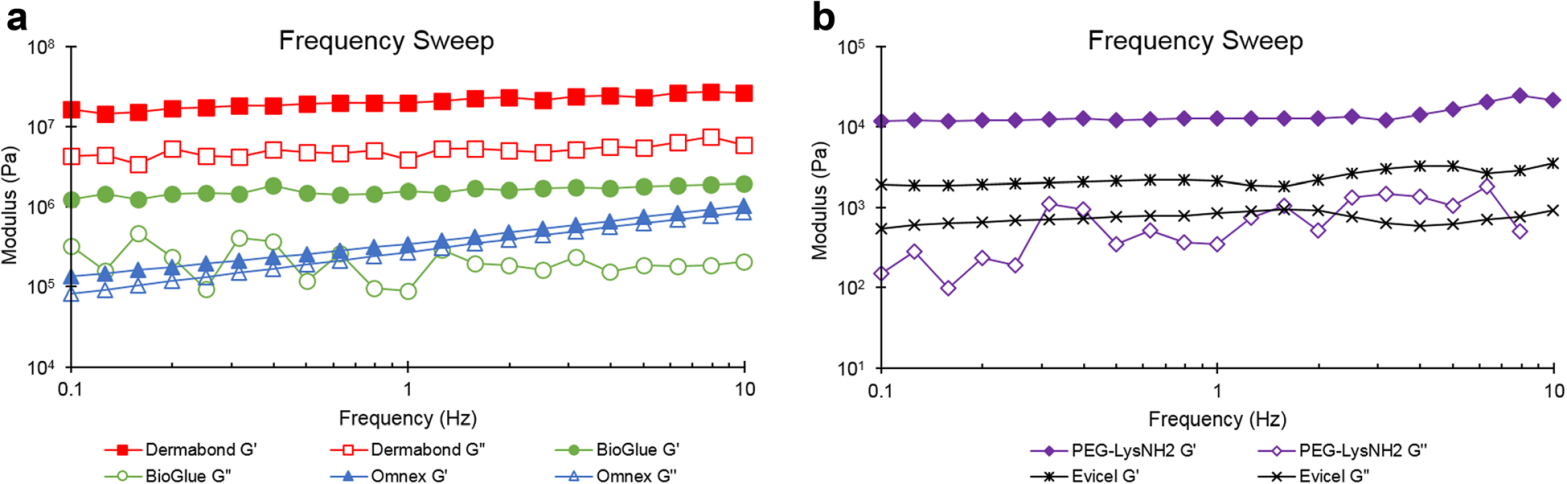
Frequency sweeps of various sealants and adhesives at 1 Hz of frequency, 50 Pa of oscillatory stress and 20°C. **a.** Dermabond, Bioglue and Omnex. **b.** Evicel and PEG-LysNH2.

Rheological measurements showed that all materials exhibited shear storage moduli (G’) greater than shear loss moduli (G”), at all frequencies and 50 Pa oscillatory stress, indicating that the samples are more elastic than viscous (Figure 3). At 1 Hz, Dermabond exhibited the highest G’ value (1.51 × 10^7^ Pa) followed by BioGlue (2.04 × 10^6^ Pa), Omnex (2.47 × 10^5^ Pa), PEG-LysNH_2_ (1.16 × 10^4^ Pa) and Evicel (3.7 × 10^3^ Pa). Samples exhibiting high G’ (e.g. Dermabond, BioGlue and Omnex) (Figure 3A) were stiffer than the samples with lower G’ (e.g. PEG-LysNH_2_ and Evicel) (Figure 3B), likely due to an increase in the cross-link density of their formed network. The loss tangent values (tan δ = G”/G’) were lower than 1 for all materials at the investigated frequency of 1 Hz (tan δ = 0.2, 0.05, 0.7, 0.04, and 0.3 for Dermabond, BioGlue, Omnex, PEG-LysNH_2_, and Evicel, respectively, at 1 Hz) with smaller tan δ values indicating a more solid-like material.

### Resistance to failure

**Figure 4:**
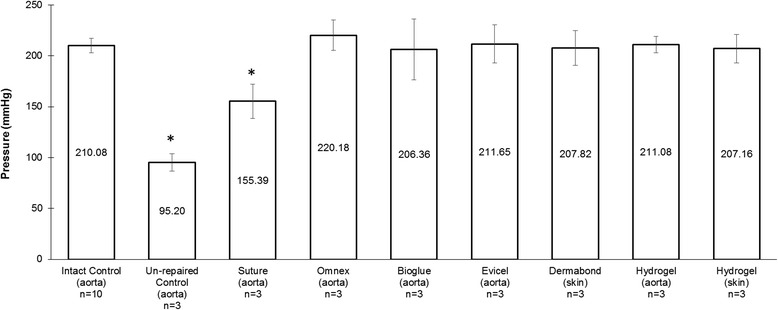
Resistance to failure**.** Pressure was gradually increased within the system and measured at 30 seconds (P_30_). Mean pressure values are reported for each group. Asterisks (*) denote statistically significant differences after ANOVA with Bonferroni correction.

When applied to 2.5 mm incisions under dry conditions, all products withstood pressures greater than 250 mmHg without signs of failure or leakage. P_30_ in sealed groups ranged between 206.36 - 220.17 mmHg and no statistically significant differences were observed between the groups (p = 0.94) (Figure 4). The control and suture groups had lower mean P_30_, when compared to sealed groups, as the pressure increase was hindered by active fluid loss (p < 0.001 for control and p = 0.013 for suture, Bonferroni-corrected α = 0.025 for 2 tests). The average relative standard error for the P_30_ measurements was 4.97 ± 1.84%.

### Endurance

**Figure 5:**
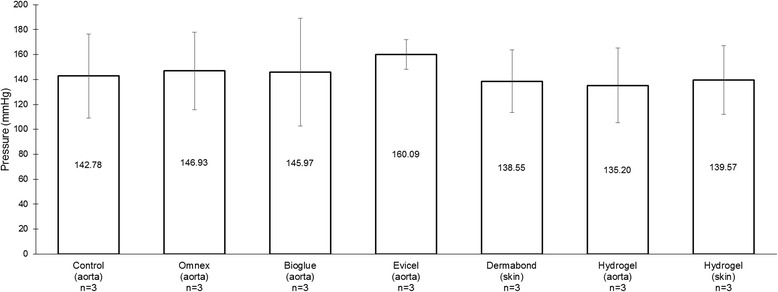
Endurance. The sealed wounds were subjected to alternating pressures for a 24-hour period. Mean pressure values are annotated for each group.

All products held the shear stresses of fluctuating pressures for a 24-hour period. Mean pressures ranged between 135.20 - 160.09 mmHg, and there were no differences between the products or between the products and the control group composed of intact tissues (p = 0.96) (Figure 5). The novel PEG-LysNH_2_ was able to seal the incisions in both *ex-vivo* vascular and dermal tissues.

## Discussion

Sealants, adhesives and hemostatic agents are useful adjuncts to surgical care, as they can reduce operative times, improve the quality of surgical tissue management, and decrease bleeding [2]. Applications for these agents include wound closure in skin and mucosal surfaces, sealing of intestinal and vascular anastomoses, prevention of cerebrospinal fluid leakage, fixation of surgical devices and grafts and hemorrhage control across all surgical specialties [2,6–8]. Products with new properties, such as the ability to deliver antimicrobial medications or to reversibly seal the wound allowing gradual re-exposure of the injury and sparing the need for debridement [5], could further reduce morbidity and improve patient outcomes.

Even though animal experimentation plays a fundamental role in the development of sealants, adhesives and hemostatic agents, the efficacy of new materials and formulations should be evaluated before proceeding to testing in animal hemorrhage models. Researchers should **replace, reduce and refine** [9,10] the use of animal models whenever possible; *ex-vivo* pressure testing offers an alternative to animal models and a means to optimize development. In order to reduce the number of animal experiments and in accordance with the guiding principles for the ethical use of animals in testing [9,10] we conducted this study in a simulated model of hemorrhage using *ex-vivo* tissues. A similar approach has been used to evaluate the strength of sealants used in the repair of cerebrospinal fluid leak [11].

The adhesives and sealants used in this study, exhibiting different dynamic mechanical strength ranging from stiff to rigid, were subsequently tested in an *ex-vivo* model of venous and arterial hemorrhage to determine their wound-closure performance. Although the materials exhibited different dynamic mechanical properties, they were able to seal *ex-vivo* injuries and withstand physiological pressures: furthermore, their efficacy was superior to that of simple suturing techniques. In particular, the PEG-LysNH_2_, a novel sealant in the initial phases of development, was included in our tests: its performance did not differ from those of previously approved sealants and adhesives, and it was also superior to simple sutures, thus confirming its sealant efficacy for small ex-vivo arterial injuries under pressures of up to 250 mmHg and for periods up to 24 hours.

An ideal surgical tissue adhesive or sealant should exhibit rapid adhesion to injured tissues and maintain strong and close apposition of wound edges for an amount of time sufficient to allow wound healing [12,13]. An ideal hemostatic agent should meet the requirements for tissue adhesives and also exhibit the following additional properties: 1) stop hemorrhage from actively bleeding vessels; 2) maintain physiological pressures for several hours; and 3) be easily applied to the tissue with a user-friendly procedure [14]. Thus far, the PEG-LysNH_2_ has demonstrated to be adherent to *ex-vivo* tissues, resistant and easily applied, but it has yet to prove its efficacy in controlling active hemorrhage in *in-vivo* models of hemorrhage. In subsequent development stages, and in order to evaluate the efficacy of the PEG-LysNH_2_ as a hemostatic agent, we will assess its efficacy in a well-established small animal model of uncontrolled hemorrhage [15], and, if successful, proceed to a large animal model: the logical steps in the design or evaluation of new hemostatic agent formulations.

Several limitations are evident when testing the efficacy of the PEG-LysNH_2_ using the proposed *ex-vivo* model. As it is presented in this study, the model is unable to assess the efficacy of hemostatic agents, as their basic mechanism of action relies on the activity of the clotting cascade. Additionally, physiologic outcomes such as survival, mean arterial pressure resuscitation fluid usage are often used to evaluate hemostatic agent efficacy [16]. To date, no *ex-vivo* model has been able to simulate the actual pathophysiologic response to injury and stress, making animal models irreplaceable in subsequent stages of product development. Therefore, with this study’s results it is only valid to conclude that the PEG-LysNH_2_ meets the basic requirement of a tissue adhesive or sealant: it physically adheres to and seals injured tissues, thereby preventing fluid loss in a physiologic pressure range.

## Conclusion

In conclusion, all the products evaluated in this study - including the novel PEG-LysNH_2_ - were able to seal small incisions in *ex-vivo* skin and vascular tissues with better outcomes than simple suture repairs. These encouraging preliminary results on the efficacy of the PEG-LysNH_2_ show that it meets the basic requirements of tissue adhesives and sealants. In order to justify its use as a hemostatic agent in emergent care scenarios, further research regarding the performance of this novel sealant in *in-vivo* models of uncontrolled hemorrhage is necessary.
